# Prevalence of anemia in diabetes mellitus in South Asia: A systematic review and meta-analysis

**DOI:** 10.1371/journal.pone.0285336

**Published:** 2023-05-10

**Authors:** Hoimonty Mazumder, Kazi Faria Islam, Farzana Rahman, Easter Protiva Gain, Nobonita Saha, Irfath Sharmin Eva, Md Monir Hossain Shimul, Jyoti Das, M. Mahbub Hossain

**Affiliations:** 1 Research Initiative for Health Equity (RiHE), Khulna, Bangladesh; 2 Institute of Nutrition and Food Science, University of Dhaka, Dhaka, Bangladesh; 3 United Nation’s Children Fund (UNICEF) Bangladesh, Ukhiya, Bangladesh; 4 Gonoshasthaya Samaj Vittik Medical College, Mirzanagar, Savar, Dhaka, Bangladesh; 5 North South University, Dhaka, Bangladesh; University of Buea, CAMEROON

## Abstract

**Objective:**

Anemia and Diabetes Mellitus (DM) are amongst major clinical and public health challenges in South Asia that influence the progression of chronic health problems in this population. Despite a growing body of research on these problems, there is a lack synthesized evidence on the burden of anemia among people with DM in this region. This meta-analytic review was conducted to estimate the prevalence of anemia among people with DM in South Asia.

**Methods:**

A systematic search of the literature was conducted in five primary databases and additional sources up to July 29, 2022, that reported the prevalence of anemia among DM patients in any of the eight South Asian countries. Observational studies that met pre-determined eligibility criteria according to the protocol registered in PROSPERO (CRD42022348433) were included in this meta-analysis. Random effect models were used to estimate pooled prevalence.

**Results:**

Of the 40 eligible studies, 38 underwent meta-analysis representing 14,194 participants with DM. The pooled prevalence of anemia was 45% (95% CI: 37.0–54.0, *I*^*2*^ = 99.28%, p = 0.00) among diabetic people in South Asia. In sub-group analysis, the pooled prevalence of anemia was higher in females (48%, 95% CI: 37.0–60.0, *I*^*2*^ = 98.86%, p = 0.00) compared to males (39%, 95% CI: 29.0–48.0, *I*^*2*^ = 98.18%, p = 0.00). Diabetic patients with older age (≥ 50 years) reported higher pooled estimates of anemia (48%, 95% CI: 38.0–58.0, *I*^*2*^ = 99.07%) than younger age group (< 50 years) (34%, 95% CI: 21.0–47.0, *I*^*2*^ = 98.83%). In addition, we found variation in pooled prevalence estimates of anemia considering the type of DM, such as type 1 reported 2% (95% CI: 0.00–4.00), type-2 reported 48% (95% CI: 40.0–56.0, *I*^*2*^ = 98.94%), and Gestational diabetes mellitus (GDM) reported 6% (95% CI: 3.00–12.0).

**Conclusion:**

High pooled estimates of anemia among diabetic patients in South Asia, including publication bias, warrants further clinical and public health research following standard research methods to understand the more context-specific epidemiological insights and evidence.

## 1. Introduction

Diabetes is a chronic metabolic syndrome manifested by hyperglycemia, which eventually can cause serious macro and microvascular complications [1]. It is highly associated with premature mortality, morbidity, and disability, impacting people’s overall quality of life. Globally, 537 million people were estimated to have Diabetes Mellitus (DM) in 2021, projecting to rise to 783 million by 2045 [2]. Diabetes disproportionately affects low-and middle-income countries, comprising 80% of the total disease burden [3]. South Asia- home to one-fourth of the world’s population, became a hotspot with a high prevalence of DM, ranging from 8.7 percent in Nepal to 30.8 percent in Pakistan [4]. Three South Asian countries- India, Pakistan, and Bangladesh were ranked within the top ten list for having the highest number of diabetic adults [5]. Many transitions, such as socioeconomic, dietary, and lifestyle, including rapid urbanization, and technological upsurge, pre-disposing the South Asian population at risk of developing diabetes and associated co-morbidities [6].

Anemia is a hematological condition with a global prevalence of 22.8%, while Africa and South Asia were identified as the regions with the highest anemia burden [7]. The concurrence of anemia and diabetes is recognized as a major public health concern, increasingly affecting the overall health status of the patients. The frequency of anemia and diabetes comorbidities ranges from 14% to 45% in the population with different ethnicities worldwide [8]. Prior studies found anemia two times more likely among diabetic patients than non-diabetics [9, 10]. However, the etiology of anemia in diabetes is multifactorial yet poorly understood. The possible underlying mechanisms include abnormal red blood cells, oxidative stress, and sympathetic denervation of the kidney resulting from hyperglycemia, which promote hypoxia and erythropoietin stress [8, 11] that eventually may cause anemia. Several characteristics in diabetic patients are found to be associated with anemia prevalence, such as age, duration of diabetes, glomerular filtration rate (GFR), nutrition, blood glucose control, and proteinuria [1, 12, 13].

The dual burden of anemia and DM are challenging from the public health perspective. Evidence suggests that people with DM may have a high anemia prevalence, and chronic anemia can be associated with DM-related complications [1, 12, 14]. Similar evidence was demonstrated in the African region with a 35% pooled prevalence of anemia among diabetic patients, and when it comes to patients with diabetic foot lesions, the prevalence went much higher. Since anemia often appears early in the progression of diabetic complications, early detection and treatment could help prevent diabetes-related vascular complications and improve their quality of life. Several South Asian countries report anemia in diabetes; however, the epidemiological evidence regarding anemia among people with DM in South Asia is minimal. To address this knowledge gap, we conducted a quantitative systematic review and meta-analysis examining anemia prevalence among patients with DM in South Asia.

## 2. Methods

### 2.1. Study design

This meta-analysis was conducted adhering to the Preferred Reporting Items for Systematic Reviews and Meta-analysis (PRISMA) (S1 Checklist) [15]. The protocol of this review has been registered in PROSPERO (registration number: CRD42022348433).

### 2.2. Definition of anemia and diabetes mellitus

Anemia is a condition characterized by reduced number of red blood cells or low levels of hemoglobin, leading to diminished ability of the blood to transport oxygen to the body’s tissues [16]. Type-1 diabetes mellitus is an autoimmune disease results from destruction of pancreatic β-cells, which is characterized by inability of pancreas to produce insulin [17–19]. Type-2 diabetes mellitus is a complex condition generally characterized by insulin resistance and the resulting hyperglycemia [19]. Gestational DM is defined as presence of any degree of glucose intolerance that is first detected during pregnancy [20]. Any form of anemia based on visual observation or clinical findings or objectively verified findings, such as hemoglobin concentration (as defined by authors) reported in studies, was considered in this review. Similarly, diagnosis of diabetes based on clinical history, previous diagnosis, or any diagnostic criteria (as defined by authors), such as blood-glucose level and HbA1c, were considered.

### 2.3. Data source and search strategy

We systematically searched five electronically databases–Medline, American Psychological Association (APA) PsycInfo, Academic Search Ultimate, Cumulative Index to Nursing and Allied Health Literature (CINAHL), and Web of Sciences for scholarly articles. The search query was organized using MeSH terms (where applicable), and appropriate set of keywords applied with Boolean operators (i.e., “OR”, “AND”) and retrieved articles from respective databases (Table 1). The detailed search strategies for all five databases are provided in the supplementary section (S1 File). The preliminary search was updated on July 29, 2022. We additionally searched Google Scholar from backward reference searching (screening references cited in the included articles) and forward citation chaining (screening papers that cited the included articles).

**Table 1:** Literature search strategy used in this systematic review and meta-analysis.

Search query	Search topic	Search keywords (titles, abstracts, and subject headings with Boolean operators)
1	Outcome of interest	"Anemia" OR “Anaemia” OR “hematological parameters” OR “red blood cell parameters”
2	Population of interest	“Diabetes Mellitus” OR “Diabetic” OR “Diabetic patient” OR “Glucose tolerance test” OR “Glucose intolerance” OR “Hyperglycemia” OR “Blood glucose” OR “Gestational Diabetes” OR “Impaired glucose tolerance” OR “Diabetes*”
3	Epidemiological phenomenon	“Prevalence” OR “Prevalence*” OR “Incidence” OR “associated factors” OR “Determinant factors” OR “Determina*” OR “Epidemiology” OR “Epidemiology*” OR “Disease burden” OR “Frequency*” OR “Magnitude”
4	Population of interest	“Afghan*” OR “Bangladesh*” OR “Bhutan*” OR “India*” OR “Maldiv*” OR “Nepal*” OR “Pakistan*” OR “Sri Lanka*” OR “South Asia”
Final search query	Intersection of four topics	1 AND 2 AND 3 AND 4

### 2.4. Eligibility criteria

We included articles that met the following criteria:

- observational studies i.e., cross-sectional, case-control, and cohort studies by design.
- reported prevalence/incidence of anemia (any kind of anemia) amongst persons with Diabetes Mellitus (Type-1 DM, Type-2 DM, and gestational DM)
- studies conducted in any of eight countries (Bangladesh, India, Maldives, Bhutan, Afghanistan, Pakistan, Nepal, and Sri Lanka) in South Asia.
- articles published in peer-reviewed journals in selected databases by English language.

We excluded articles that did not comply with any of these primary criteria. Case reports, case series, letters to editor, commentaries, protocols, conference proceeding or abstract, and review papers were excluded from this review. Moreover, preprints were excluded since they did not go through the peer-review process.

### 2.5. Study selection

We used a cloud-based systematic review management portal (rayaan.ai) to evaluate all retrieved citations. Two authors independently screened the title and abstract of retrieved articles following removal of duplicates. At the end of primary screening, a third author resolved any conflicts in terms of eligibility of citation through discussion. Then, articles appeared to be eligible and underwent full-text review, data extraction, and subsequent analyses.

### 2.6. Data extraction

We have created a template in Microsoft Excel to capture data from the finally recruited articles. Data on the following variables were extracted: author information, year of publication, study design and settings, sample size, study population characteristics, diagnostic cut-off value of hemoglobin and prevalence of anemia in the respective study. Two authors independently extracted data from each of the included articles. Then, these two separate datasets were evaluated by a third author to resolve potential inconsistencies at the end of the data extraction.

### 2.7. Quality appraisal of the included studies

In this review, we used JBI critical appraisal checklist for prevalence using 9 criteria [21], which has been used in similar meta-analyses previously [1, 12]. Three reviewers independently assessed the study quality and then resolved potential inconsistencies through discussion. Finally, based on their mean score we graded them as high (0–4), moderate (5–6), and low (7–9) risk of bias.

### 2.8. Statistical analyses

Statistical analyses were performed to estimate the pooled prevalence of anemia in diabetes mellitus using a random-effect model with DerSimonian and Laird transformed inverse variance method in STATA SE 17.0 software [22, 23]. Heterogeneity across the studies was assessed using Cochrane chi-square at a significant level of p <0.1, and *I*^*2*^ value. Hence, *I*^*2*^ statistic is categorized as low (25%-50%), moderate (51%-75%), or high (>75%) [24].

### 2.9. Subgroup analyses

Subgroup meta-analyses were conducted for studies reporting anemia by age (<50 years vs ≥ 50 years), gender (male vs. female), duration of having DM (less than 5 years vs 5 years and over), type of DM, population with diabetic complications, study design, country, publication year, sample size, study settings, and risk of bias to identify between-group variations including the possible sources of heterogeneity.

### 2.10. Sensitivity analyses

Sensitivity analyses were performed to quantify the impact of individual study on the overall pooled estimates and calculated pooled prevalence for rest of the studies after excluding each one.

### 2.11. Meta-regression analyses

Meta-regression was performed to investigate the association between the potential covariates in studies and pooled prevalence of anemia among Diabetic population. We used mean age, country, sample size, study settings, type of DM, and risk of bias in meta-regression analyses to assess their association with pooled estimates.

### 2.12. Publication bias

The visual inspection of forest plot and performing Egger’s regression test were undertaken to evaluate publication bias which may affect the generalizability of the study results. A p-value < 0.05 in Egger’s test was considered as evidence that publication bias exists [25].

## 3. Results

**Fig 1:**
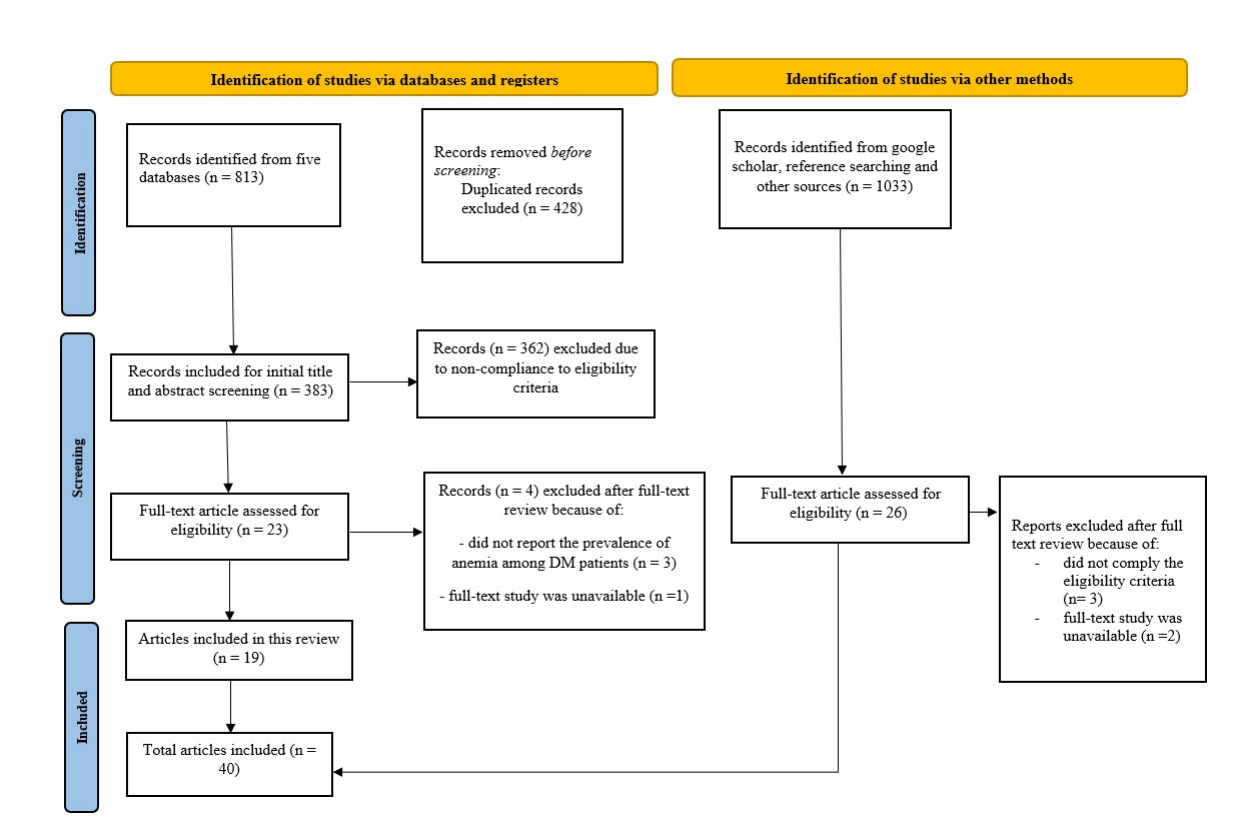
Flow diagram of the literature retrieval process.

A total of 813 articles were retrieved from selected electronic databases, from which 428 duplicates were removed. We found 23 citations that underwent full-text evaluation following preliminary title and abstract screening using pre-determined eligibility criteria. From additional sources, another 26 citations went through full-text evaluation. A PRISMA flowchart illustrating the detailed literature search process is shown in (Fig 1). Finally, a total of 40 articles were included in this systematic review [26–66].

### 3.1 Characteristics of the included studies

The summary overview of the included articles is provided in “Table 2”. The vast majority of the included studies were conducted in India (n = 34) followed by Pakistan (n = 5), and Bangladesh (n = 1). We found no studies from Afghanistan, Bhutan, Maldives, Nepal, and Sri Lanka. All studies were observational, 34 (85%) studies utilized cross-sectional study design, 5 (13%) studies were case-control and 1 (3%) was cohort in design. Most of the studies (n = 36, 90%) were conducted in hospital settings, and only a few (n = 4, 10%) were community-based. Of hospital-based studies, 18 recruited patients from the outpatient department (OPD), 4 from the in-patient department (IPD), and 14 had patients either from both OPD and IPD or un-specified. The sample size of the diabetic population varied across the studies, ranging from 48 to 6000. About 85% (n = 34) of included studies enrolled Type-2 diabetic patients, whereas only 2 studies recruited Type-1 diabetes mellitus and one study with mixed Type-1 and Type-2 DM and another one with gestational diabetic (GDM) patients. The mean age of the participants was 49 years (95% CI: 46.0–56.32 years). The duration of illness among DM patients across the included studies ranged from <1 to 13.06 years. There were a small number of studies that reported anemia among patients with diabetes complications, such as diabetic nephropathy (n = 5), retinopathy (n = 5), and neuropathy (n = 1). To determine anemia, studies (n = 29) mainly applied World Health Organization (WHO) guidelines that categorized anemia if hemoglobin level <13 g/dl for males and <12 g/dl for females. Most studies (n = 28) were published before 2020.

**Table 2:** Descriptive summary of included studies on the prevalence of anemia in DM patients in South Asia (n = 40).

Authors and publication year	Country	Study design and settings	Population characteristics	Mean age ± SD/ age range; Type of DM; Duration of DM	Sample size of Diabetic population (Male, Female)	Diagnostic cut-off value of hemoglobin to determine anemia	Prevalence anemia (%)
Ahmed et al., 2017 [26]	Karachi, Pakistan	Cross-sectional; Hospital	Patients with type-2 DM over 20 years of age	Mean age: 56.5 ± 10.5 years (age range: 20–80 years); Type-2 DM; Median duration: 4 years.	640 (M: 340, F: 300)	World Health Organization definition, i.e., hemoglobin level <13 g/dl in men and <12 g/dl in women to define anemia	41.71
Ahmed et al., 2013 [27]	Bangalore, India	Prospective cross-sectional study; Hospital	Diabetic patients with age >18 years and >5 years duration of DM	Age range: 21—over 80 years (greater proportion belongs to age over 50 years); Tyep-2 DM; More than 5 years.	120 (M: 73, F: 47)	NR	56.70
Arshad et al., 2021 [30]	Karachi, Pakistan	Cross-sectional; Hospital	Diabetic patients above 18 years of age and diagnosed as DM by clinical or laboratory reports.	Mean age: 54 years (age range: 18-over 60 years); Type-2 DM; Not specified.	277 (M: 121, F: 156)	Female; Mild: 11.0–11.9 g/dl, Moderate; 8.0–10.9 g/dl, Severe: < 8.0 g/dl. Male; Mild: 11.0–12.9 g/dl, Moderate: 8.0–10.9 g/dl, Severe: < 8.0 g/dl.	80.0
Baisakhiya et al., 2017 [32]	Haryana, India	Case-control study; Hospital	Patients diagnosed having Type-2 DM with retinopathy were recruited as Case and Type-2 DM without retinopathy as Control.	Mean age of diabetic subjects without retinopathy was 62.3 ± 0.98 years and with retinopathy was 65.78 ± 0.56 years; Type-2 DM; Not specified.	90 (M: 44, F: 46)	World Health Organization definition, i.e., hemoglobin level <13 g/dl in men and <12 g/dl in women to define anemia	32.20
Hafdhallah et al., 2017 [28]	Gujrat, India	Cross-sectional; Hospital	Half of the total participants were recruited those having Type-2 DM with nephropathy and other half Type-2 DM without nephropathy	Over 45 years; Type-2 DM; Not specified.	170	World Health Organization definition, i.e., hemoglobin level <13 g/dl in men and <12 g/dl in women to define anemia	66.0
Babu et al., 2013 [31]	West Bengal, India	Cross-sectional; Hospital	Type-2 DM with diabetic neuropathy and free from retinopathy and albuminuria	Age range: 30 to over 60 years (greater proportion belongs to over 50 years); Type-2 DM; Not specified.	100	World Health Organization definition, i.e., hemoglobin level <13 g/dl in men and <12 g/dl in women to define anemia	55.0
Bharathi et al., 2016 [33]	Chennai, India	Case-control study; Hospital	Type-2 DM	NR; Type-2 DM; 5 years or more.	100 (M: 100)	World Health Organization definition, i.e., hemoglobin level <13 g/dl in men and <12 g/dl in women to define anemia	26.0
Gutch et al., 2015 [35]	Uttar Pradesh, India	Cross-sectional; Hospital	Children and adolescent aged <18 years	Mean age: 11.5 ± 6.4 (age range: 2.5 years to 18 years); Type-1 DM; Duration of Diabetes varies from newly diagnosed to 12 years.	164 (M: 90, F: 74)	NR	1.20
Chowdeswari et al., 2016 [34]	Nellore, India	Retrospective cross-sectional study; Hospital	Type-2 DM	Mean age: 32.7± 1.8 (age range: 18–70 years); Type-2 DM; Not specified.	1000 (M: 358, F: 642)	Mild anemia was categorized as hemoglobin levels of 12–12.9 g/dL in males and 11–11.9 g/dL in females; moderate anemia with 9–11.9 g/dL in males and 8–10.9 in females; severe was <9g/dl in males and <8 g/dL in females	16.30
Hussain et al., 2019 [37]	New Delhi, India	Cross-sectional; Hospital	Type-2 DM with any stages of chronic kidney disease (CKD)	Mean age: 56 ± 11.25 years; Type-2 DM; Mean duration: 9.6 ± 4.57 years.	323 (M: 156, F: 167)	World Health Organization definition, i.e., hemoglobin level <13 g/dl in men and <12 g/dl in women to define anemia	70.27
Ansari et al., 2020 [29]	Hydrabad, India	Cross-sectional; Hospital	Known Diabetic menstruating and menopausal women	NR; Type-2 DM; Not specified.	120 (F: 120)	NR	60.83
Jayalakhshmi et al., 2012 [38]	Bangalore, India	Cross-sectional; Hospital	Type-2 DM	Mean age: 55.87 years; Type-2 DM; Duration: <4 to >8 years.	205 (M: 125, F: 80)	World Health Organization definition, i.e., hemoglobin level <13 g/dl in men and <12 g/dl in women to define anemia	47.80
Joshi et al., 2015 [39]	Mumbai, India	Cross-sectional; Hospital	Children and adolescent age 0–18 years diagnosed Type-1 DM, who receiving insulin therapy	Age range =: 0–18 years; Type-1 DM; Mean duration: 8.2 ± 5.6 years.	71	World Health Organization definition, i.e., hemoglobin level <13 g/dl in men and <12 g/dl in women to define anemia	25.40
Karoli et al., 2013 [40]	Uttar Pradesh, India	Cross-sectional; Hospital	Type-2 DM and normoalbuminuric who were referred for screening of Diabetic Retinopathy.	Mean age: 56 ± 12 years; Type-2 DM; Mean duration: 8.2 ± 5.6 years.	226 (M: 110, F: 116)	NR	27.63
Shah et al., 2018 [41]	Mumbai, India	Retrospective study; Hospital	Type-2 DM aged > = 40 years	Mean age: 65.2 ± 25.1 years; Type-2 DM; Mean duration: 11.9 ± 6.9 years.	6000 (M: 3498, F: 2502)	NR	56.47
Manglunia et al., 2018 [42]	Jaipur, India	Cross-sectional; Hospital	Type-2 DM patients	Mean age: 60.33± 14.18 in anemia group; Type-2 DM; Mean duration: 13.06 ± 10.13 in anemic group.	120 (M: 75, F: 45)	World Health Organization definition, i.e., hemoglobin level <13 gm/dl in men and <12 gm/dl in women to define anemia	66.67
Mohan et al., 2011 [43]	Bangalore, India	Cross-sectional; Hospital	Type-2 DM patients	Mean age: 56.41 ± 10.1 years (age range: 32–85 years); Type-2 DM; Mean duration: 7.59 ± 6.3 years.	306 (M: 193, F: 113)	NR	57.30
Muhammad et al., 2020 [44]	Sindh, Pakistan	Case-control study; Hospital	Type-2 DM patients without renal, hematological, or chronic liver disease	Mean age: 56.2 ± 8.01 years; Type-2 DM; Not specified	106 (M: 54, F: 52)	World Health Organization definition, i.e., hemoglobin level <13 gm/dl in men and <12 gm/dl in women to define anemia	40.60
Panda et al., 2018 [45]	Maharashtra, India	Cross-sectional; Hospital	Type-2 DM with or without renal insufficiency	Mean age: 51.39 ± 8.8 years; Type-2 DM; Not specified	54 (M: 33, F: 21)	World Health Organization definition, i.e., hemoglobin level <13 gm/dl in men and <12 gm/dl in women to define anemia	63.0
Paul et al., 2017 [46]	Bangladesh	Case-control; Hospital	Type-2 DM were categorized into groups based on patients with controlled diabetes and those with poorly controlled diabetes	Mean age: 43 ± 11 years (age range 30–60 years); Type-2 DM; Not specified	105 (M: 51, F: 54)	World Health Organization definition, i.e., hemoglobin level <13 gm/dl in men and <12 gm/dl in women to define anemia	53.0
Praveen et al. 2020 [48]	Delhi, India	Cross-sectional; Hospital	Type-2 DM with age group of more than 20 years	Mean age: 49.76 ± 10.38 in patients with total iron deficiency anemia; Type-2 DM; Not specified	89	World Health Organization definition, i.e., hemoglobin level <13 gm/dl in men and <12 gm/dl in women to define anemia	16.80
Raman et al., 2011 [49]	Chennai, India	Cross-sectional; Community	Type-2 DM selected from general population, both known and newly diagnosed	Age range: 30–76 years (greater proportion of population belongs to over 40 years); Type-2 DM; Mean duration: 10.96 ± 8.31 amongst ≤ 40 years age group and 4.69± 5.44 amongst >40 years age group.	1414 (M: 750, F: 664)	World Health Organization definition, i.e., hemoglobin level <13 gm/dl in men and <12 gm/dl in women to define anemia	12.30
Raman et al., 2012 [50]	Chennai, India	Cross-sectional; Community	Type-2 DM selected from general population, both known and newly diagnosed	Mean age: 56.32+ 10.02 years (age range: 40–67 and over); Type-2 DM; Mean duration: 10.96 ± 8.31 amongst ≤ 40 years age group and 4.69± 5.44 amongst >40 years age group.	1414 (M: 750, F: 664)	World Health Organization definition, i.e., hemoglobin level <13 gm/dl in men and <12 gm/dl in women to define anemia	12.30
Rani et al., 2010 [51]	Chennai, India	Cross-sectional; Community	Type-2 DM selected from general population, both known and newly diagnosed	Age range: 40 to over 69 years (greater proportion belongs to 50 years and over); Type-2 DM; Duration of DM: greater proportion belongs to over 5 years	1414 (M: 750, F: 664)	World Health Organization definition, i.e., hemoglobin level <13 gm/dl in men and <12 gm/dl in women to define anemia	12.30
Rathod et al., 2016 [52]	Gujrat, India	Cross-sectional; Hospital	Patients with Type-2 DM aged more than 30 years	Mean age: 58 ±14 years in male and 62 ±12 years in female; Type-2 DM; Not specified.	200 (M: 114, F: 86)	World Health Organization criteria for Anemia	18.0
Rathod et al., 2018 [53]	Gujrat, India	Cross-sectional; Hospital	Patients with Type-2 DM aged more than 20 years	Age range: 24–72 years (greater proportion belongs to over 51 years) Type-2 DM; Not specified.	100 (M: 66, F: 34)	Definition for anemia hemoglobin values <13.0 g/dl for men and <12.0 g/dl for women.	44.0
Reddy et al., 2021 [54]	Bangalore, India	Cross-sectional; Hospital	Patients of either sex aged between 18–60 years, who presented Type-2 DM for more than 5 years on medication with HbA1c more than 6.5% including no renal impairment.	Mean age: 53 years (age range: 18–60 years); Type-2 DM; Mean duration of DM among anemic diabetes was 10.5 years	150 (M: 71, F: 79)	World Health Organization definition, i.e., hemoglobin level <13 gm/dl in men and <12 gm/dl in women to define anemia	57.33
Reddy et al., 2019 [55]	Andhra Pradesh, India	Cross-sectional; Hospital	Type-2 DM patients	Mean age: 61.56 ± 0.127; Type-2 DM; Not specified	100 (M: 50, F: 50)	Anemia, as defined by World Health Organization (WHO) criteria less than 130 g/L for men and less than 120 g/L for women	43.0
Sajid et al., 2020 [56]	Lucknow, India	Cross-sectional; Hospital	Both male and females aged 30–60 years,	Mean age: 44.17 ± 8.82; Type-2 DM; Not specified.	48 (M: 21, F: 27)	NR	47.92
Newtonraj et al., 2019 [64]	Maharashtra, India	Cross-sectional; Community	Diagnosed Type-2 DM	NR; Type-2 DM; Not specified	201 (M: 75, F: 126)	World Health Organization definition, i.e., hemoglobin level <13 gm/dl in men and <12 gm/dl in women to define anemia	69.15
Shabeeb et al., 2021 [66]	Karnataka, India	Prospective cross-sectional study; Hospital	Type-2 DM patients	Age range: 30–79 years (greater proportion of total population belongs to less than 50 years); Type-2 DM; Not specified	150 (M: 73, F: 77)	World Health Organization definition, i.e., hemoglobin level <13 gm/dl in men and <12 gm/dl in women to define anemia	65.30
Shams et al., 2015 [57]	Karachi, Pakistan	Cross-sectional; Hospital	Type-1 and Type -2 DM aged > 18 years of either gender	Mean age: 51 ± 12.4 (age range: 18–85 years); Type-1 and Type-2 DM; Mean duration: 7.6 ± 5.5 years	130 (M: 34, F: 96)	World Health Organization definition, i.e., hemoglobin level <13 gm/dl in men and <12 gm/dl in women to define anemia	63.0
Sharif et al., 2014 [58]	Karachi, Pakistan	Cross-sectional; Hospital	Type-2 DM patients	Age range: above 40 years; Type-2 DM; Not specified	200 (M: 100, F: 100)	World Health Organization definition, i.e., hemoglobin level <13 gm/dl in men and <12 gm/dl in women to define anemia	63.0
Srinivasa et al., 2014 [59]	Bangalore, India	Cross-sectional; Hospital	Either newly detected Gestational Diabetes Mellitus (GDM) patients or on follow-up; age between 18–35 years.	Mean age: 26.09 ± 3.4 years (age range: 18–35 years); GDM; Not specified	100 (F: 100)	NR	6.00
Sruthi et al., 2021 [65]	Tamil Nadu, India	Cohort study; Hospital	Patients with confirmed diagnosis of Type-2 DM with established or recently diagnosed clinical evidence of Diabetic retinopathy (DR)	Mean age: 56.68+10.1 years; Type-2 DM; Mean duration of DM: 2.9 ± 1.0 for mild NPDR, 6.5 ± 1.8 for moderate NPDR, 8.5 ± 3.3 for severe NPDR and 15.8 ± 1.4 for PDR.	240 (M: 130, F: 110)	Hemoglobin levels <13g/dl in males and <12g/dl in females were considered as Anemic	40.40
Swarnkar et al., 2015 [63]	U.P., India	Cross-sectional; Hospital	Type-2 DM patients with normal serum creatinine levels and creatinine clearance <90 ml	Mean age: 52.38 ± 11.26 years (age range: 20–78 years); Type-2 DM; Not specified	200 (M: 90, F: 110)	World Health Organization definition, i.e., hemoglobin level <13 gm/dl in men and <12 gm/dl in women to define anemia	40.00
Umeshchandara et al., 2021 [62]	Bangalore, India	Cross-sectional; Hospital	Type-2 DM with normal renal function	Mean age: 54.11 ± 12.14 years; Type-2 DM; Duration: less than 1 to 10 years and over.	230 (M: 54, F: 176)	NR	21.73
Valarmathil et al., 2018 [60]	Tamil Nadu, India	Prospective cross-sectional; study; Hospital	Half of the participants were with better glycemic control and other half were poor glycemic control	NR; Type-2 DM; Not specified	60	NR	65.00
Wali et al., 2022 [61]	Karnataka, India	Cross-sectional; Hospital	Type-2 DM patients	Mean age: 48.59 ± 7.5; Type-2 DM; Mean duration: 3.75 ± 1.8 years	215 (M: 90, F: 125)	World Health Organization definition, i.e., hemoglobin level <13 gm/dl in men and <12 gm/dl in women to define anemia	41.86
Kumar et al., 2017 [36]	Karnataka, India	Case-control; Hospital	Type-2 DM patients having treatment	Mean age: 55.7 ± 3.6 years; Type-2 DM; Duration: Over 5 years.	70 (M: 30, F: 40)	WHO guideline for Anemia	71.40

NR = Not reported

Following quality assessment using JBI critical appraisal checklist, we identified 7 studies with low, 11 with moderate, and 22 with a high risk of bias (S1 Table). In addition, we performed a meta-analysis to estimate the pooled prevalence of anemia among the population with DM in South Asia, including sub-group estimation, sensitivity analyses, meta-regression, and publication bias.

### 3.2 Prevalence of anemia among population with diabetes mellitus in South Asia

#### 3.2.1 Pooled prevalence of anemia among diabetic people

**Fig 2:**
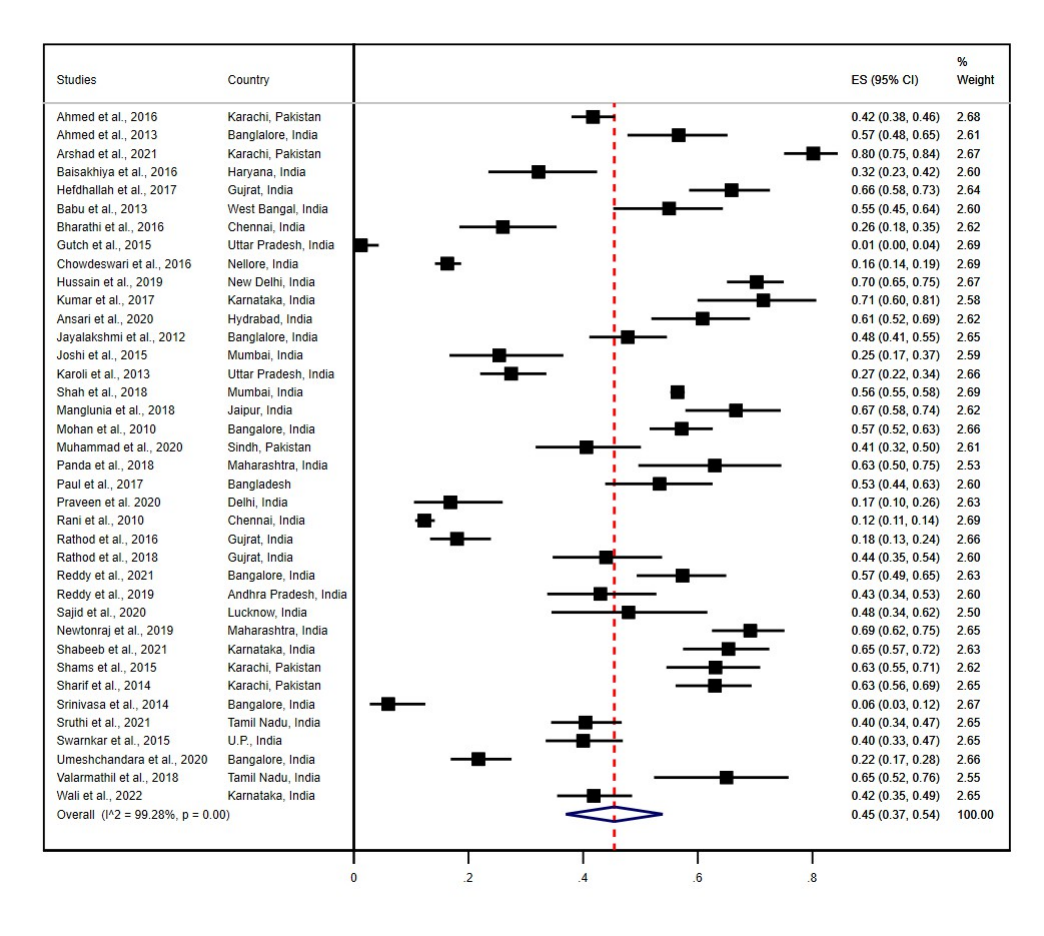
Forest plot showing the pooled prevalence of anemia among diabetes mellitus patients in South Asia.

Of the total, 38 studies that reported the prevalence of anemia among diabetes population in South Asian countries were included in this meta-analytic model. Three studies used the same sample population to report anemia in DM [49–51]; we included one of them [51]. The overall pooled prevalence of anemia was 45% (95% CI: 37.0–54.0) among 14194 participants with DM in 38 studies. We have assessed the heterogeneity of included studies using *I*^*2*^ statistics, which was found statistically significant (*I*^*2*^ = 99.28%, p = 0.00) (Fig 2).

#### 3.2.2 Meta-regression and subgroup analysis

Since there was high heterogeneity in the meta-analysis, we conducted sub-group analysis and meta-regression to evaluate the potential source of heterogeneity and correlations between the study-level explanatory variables and pooled estimate. The prevalence of anemia among people with DM was not significantly associated with country context (p = 0.156), study design (p = 0.970), sample size (p = 0.439), type of DM (p = 0.281), study settings (p = 0.720), and risk of bias (p = 0.332) (S2 Table). Only the mean age (p = 0.002) of the participants was found to be significantly correlated with pooled anemia prevalence.

In sub-group analysis, we showed variations in the pooled estimates considering different groups of the study characteristics (Table 3). We stratified the sample population based on the type of DM recruited in studies. The pooled prevalence of anemia was 63% among combined type-1 and type-2 DM (95% CI: 55.0, 71.00, n = 1), followed by 48% among type-2 DM (95% CI: 40.0–56.0, *I*^*2*^ = 98.94%, n = 34), 2% among Type-1 DM (95% CI: 0.00–4.00, n = 2) and 6% (95% CI: 3.00–12.0, n = 1) among gestational DM patients. Female participants showed a higher prevalence of anemia (48%, 95% CI: 37.0–60.0, *I*^*2*^ = 98.18%, n = 23) compared to male participants (39%, 95% CI: 29.0–48.0, *I*^*2*^ = 98.86%, n = 21). Considering the mean age or age range of greater proportion of the sample population, we categorized study populations into two groups- less than 50 years and 50 years or more. Hence, diabetic patients aged 50 years or more reported pooled prevalence of anemia of 48% (95% CI: 38.0–58.0, *I*^*2*^ = 99.07%, n = 10), whereas patients aged less than 50 years reported pooled anemia prevalence of 34% (95% CI: 21.0–47.0, *I*^*2*^ = 98.83%, n = 23). Since 37% (n = 14) studies specifically reported duration of having DM among study participants, those having DM 5 years or more showed higher prevalence (49%, 95% CI: 34.0–64.0, *I*^*2*^ = 99.44%, n = 12) than those of having DM less than 5 years (42%, 95% CI: 38.0–45.0, n = 2). Few studies explored anemia among patients with diabetic complications and reported prevalence of 68% (95% CI: 54.0–79.0, n = 1) in diabetic neuropathy, 49% (95% CI: 16.0–83.0, *I*^*2*^ = 98.93%, n = 5) in diabetic nephropathy and 43% (95% CI: 19.0–67.0, *I*^*2*^ = 99.01%, n = 5) in diabetic retinopathy patients.

**Table 3:** Subgroup analyses of the prevalence of anemia among population with diabetes mellitus in South Asia.

Subgroup						
			No. of studies	Pooled prevalence (95% CI)	*I* ^*2*^	p-value
Country	Bangladesh		1	53% (95% CI: 44.0–63.0)	-	-
	India		32	43% (95% CI: 34.0–52.0)	99.32%	0.00
	Pakistan		5	58% (95% CI: 40.0–75.0)	97.67%	0.00
Gender	Male		21	39% (95% CI: 29.0–48.0)	98.18%	0.00
	Female		23	48% (95% CI: 37.0–60.0)	98.86%	0.00
Type of Diabetes mellitus (DM)	Type -1 DM		2	2% (95% CI: 0.00–4.00)	-	0.00
	Type-2 DM		34	48% (95% CI: 40.0–56.0)	98.94%	0.00
	Mixed Type-I & Type-II		1	63% (95% CI: 55.0–71.0)	-	-
	Gestational diabetes (GDM)		1	6% (95% CI: 3.00–12.0)	-	-
Study design	Cross-sectional		31	45% (95% CI: 36.0–54.0)	99.09%	0.00
	Case-control		6	47% (95% CI: 35.0–59.0)	94.35%	0.00
	Cohort		1	40% (95% CI: 34.0–47.0)	-	-
Sample size	Small (≤ Median)		20	48% (95% CI: 38.0–58.0)	96.21%	0.00
	Large (> Median)		18	43% (95% CI: 30.0–55.0)	99.63%	0.00
Age	<50 years		10	34% (95% CI: 21.0–47.0)	98.83%	0.00
	≥50 years		23	48% (95% CI: 38.0–58.0)	99.07%	0.00
Duration of DM	Less than 5 years		2	42% (95% CI: 38.0–45.0)	-	-
	5 years or more		12	49% (95% CI: 34.0–64.0)	99.44%	0.00
Publication year	Before 2020		28	45% (95% CI: 35.0–55.0)	99.41%	0.00
	2020 and after		10	47% (95% CI: 33.0–62.0)	97.62%	0.00
Population with diabetic complications	Diabetic retinopathy		5	43% (95% CI: 19.0–67.0)	99.01%	0.00
	Diabetic nephropathy		5	49% (95% CI: 16.0–83.0)	98.93%	0.00
	Diabetic neuropathy		1	68% (95% CI: 54.0–79.0)	-	-
Risk of bias	Low risk of bias		5	50% (95% CI: 26.0–74.0)	99.78%	0.00
	Moderate risk of bias		11	49% (95% CI: 31.0–68.0)	99.42%	0.00
	High risk of bias		22	42% (95% CI: 34.0–51.0)	96.52%	0.00
Study settings	Community		2	16% (95% CI: 14.0–18.0)	-	-
	Hospital	Overall	36	46% (95% CI: 37.0–55.0)	99.18%	0.00
		Outpatient department (OPD)	18	45% (95% CI: 37.0–52.0)	95.44%	0.00
		In-patient department (IPD)	4	47% (95% CI: 5.00–88.0)	99.50%	0.00
		Both OPD & IPD/ unspecified	14	47% (95% CI: 34.0–60.0)	99.18%	0.00

At the country level, Pakistan (58%, 95% CI: 40.0–75.0, *I*^*2*^ = 97.67%, n = 5) had the highest prevalence of anemia in DM, followed by Bangladesh (53%, 95% CI: 44.0–63.0, n = 1) and India (43%, 95% CI: 34.0–52.0, *I*^*2*^ = 99.32%, n = 5). Studies with case-control design had pooled prevalence of anemia 47% (95% CI: 35.0–59.0, *I*^*2*^ = 94.35%, n = 6) whereas Cross-sectional studies had 45% (95% CI: 36.0–54.0, *I*^*2*^ = 99.09%, n = 31) and cohort study had 40% (95% CI: 34.0–47.0, n = 1) anemia prevalence. Studies having smaller sample sizes reported a higher pooled prevalence of anemia (48%, 95% CI: 38.0–58.0, *I*^*2*^ = 96.21%, n = 20) compared to larger sample sizes (43%, 95% CI: 30.0–55.0, *I*^*2*^ = 99.63%, n = 18). Moreover, a huge variation in pooled estimates of anemia was observed in different study settings. Hospital-based studies demonstrated pooled prevalence of anemia of 46% (95% CI: 37.0–55.0, *I*^*2*^ = 99.18%, n = 36), whereas community-based studies reported 16% (95% CI: 14.0–18.0, n = 2). We found a lower prevalence of anemia in studies having a high risk of bias (42%, 95% CI: 34.0–51.0, *I*^*2*^ = 96.52%, n = 22) and showed an ascending trend with moderate (49%, 95% CI: 31.0–68.0, *I*^*2*^ = 99.42%, n = 11) and low risk (50%, 95% CI: 26.0–74.0, *I*^*2*^ = 99.78%, n = 5) of bias. Furthermore, studies published in 2020 and afterwards showed a little higher prevalence of anemia (47%, 95% CI: 33.0–62.0, *I*^*2*^ = 97.62%, n = 10) than studies published before 2020 (45%, 95% CI: 35.0–55.0, *I*^*2*^ = 99.41%, n = 28).

#### 3.2.3 Sensitivity analysis and publication bias

**Fig 3:**
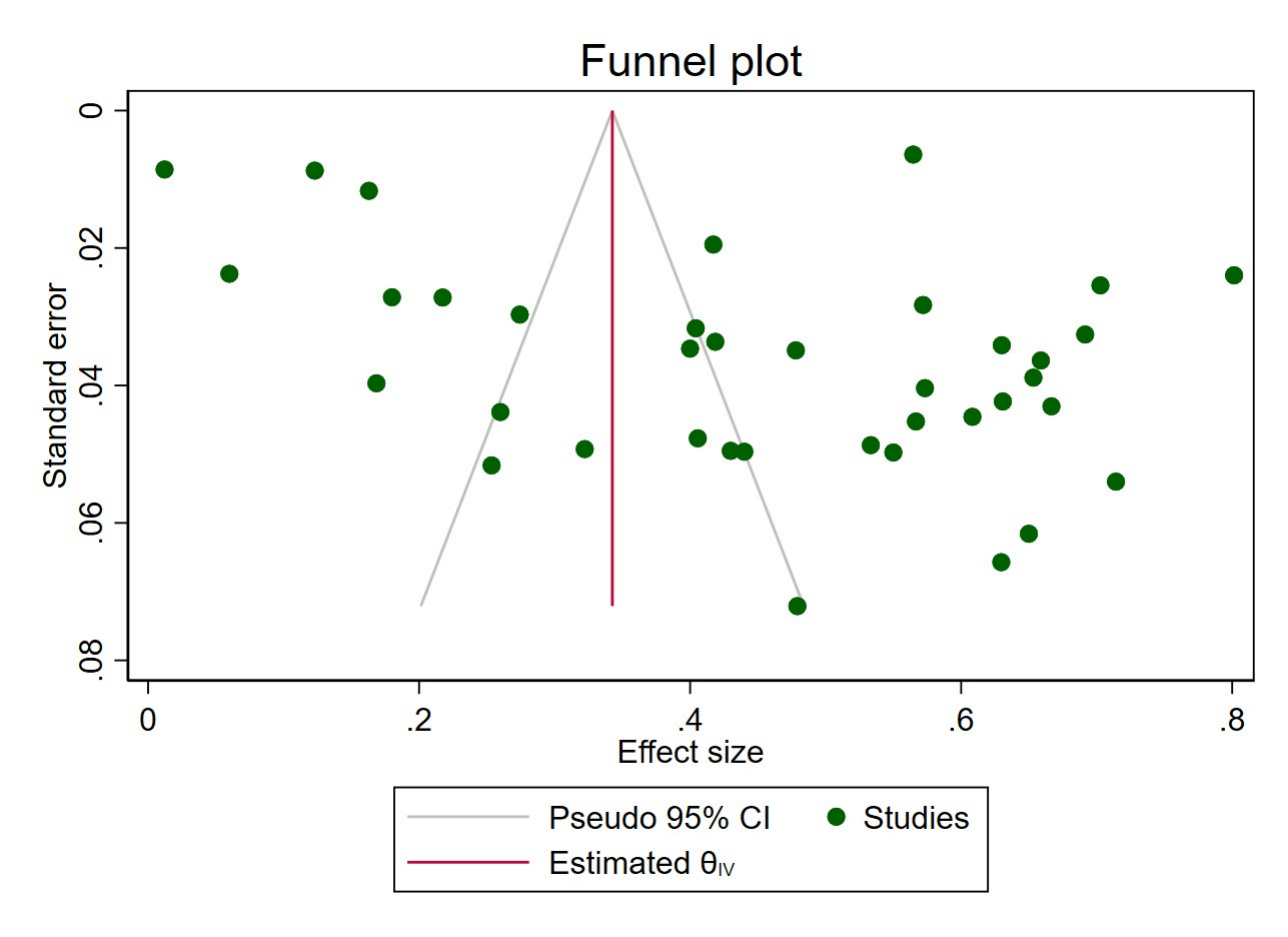
Funnel plot with pseudo 95% CI showing the publication bias of included studies on the prevalence of anemia among DM patients.

Due to high heterogeneity, we performed a sensitivity analysis by excluding each study one-by-one from the meta-analytic model. This finding showed no substantial changes in the pooled prevalence of anemia among DM patients in South Asia (S1 Fig). Furthermore, publication bias was assessed through visual inspection of the funnel plot and using a statistical regression—Egger’s test. The marked asymmetry in the funnel plot suggests publication bias (Fig 3) corroborated by Egger’s test result (Wald chi-square for residual heterogeneity = 5.79, p-value = 0.016) (S2 Fig).

## 4. Discussion

This meta-analysis was performed to quantitively synthesize the epidemiological burden of anemia among diabetic patients in South Asia. Anemia is highly prevalent in low-and middle-income countries and is a known complication of diabetes mellitus. Due to its complex and multifactorial pathogenesis, anemia may originate from various chronic diseases, including diabetes mellitus, and can contribute to aggravating disease conditions or developing complications.

The overall pooled prevalence of anemia was 45% (95% CI: 37.0–54.0) with 14194 DM patients in South Asia- which was higher than the prevalence of anemia among DM patients in Africa [14]. Patients with Type-2 DM demonstrated a higher prevalence of anemia (48%, 95% CI: 40.0–56.0, *I*^*2*^ = 98.94%) compared to type-1 (2%, 95% CI: 0.00–4.00) and gestational DM (6% (95% CI: 3.00–12.0). Female diabetic patients reported a prevalence of anemia of 48% (95% CI: 37.0–60.0), whereas males reported 39% (95% CI: 29.0–48.0). In addition, we found a higher anemia burden among diabetic patients aged 50 years or over (48%, 95% CI: 38.0–58.0, *I*^*2*^ = 99.07%) than their younger aged counterparts (34%, 95% CI: 21.0–47.0, *I*^*2*^ = 98.83%). Since the majority of studies recruited sample population from the hospital, pooled estimates of anemia in the community (16%, 95% CI: 14.0–18.0) and hospital-based settings (46%, 95% CI: 37.0–55.0, *I*^*2*^ = 99.18%) showed substantial variation in sub-group analysis. Studies with low risk of bias (50%, 95% CI: 26.0–74.0, *I*^*2*^ = 99.78%) reported a higher anemia prevalence, followed by studies with moderate (49%, 95% CI: 31.0–68.0, *I*^*2*^ = 99.42%) and high risk of bias (42%, 95% CI: 34.0–51.0, *I*^*2*^ = 96.52%).

In this study, female diabetic patients in South Asia reported a higher prevalence of anemia (48%,95% CI: 37.0–60.0) compared to their male counterparts 39% (95% CI: 29.0–48.0). However, this finding contradicts other evidence from the USA and African countries [1, 14, 67, 68]. The high historical burden of anemia among females in this region due to iron and folic acid deficiencies, including other pre-disposing factors such as poor glucose control, high metabolic risk factors, and low healthcare utilization, might explain this fact [69]. Anemia is also a serious concern among males, though often unrecognized, affecting as higher as 40% of men in India [70]. The continually increasing burden of diabetes and its related risk factors in the South Asian population largely involved in metabolic derangement may contribute to the increased risk of anemia. Older age is an important predictor of anemia and diabetes both. We found a higher prevalence of anemia among DM patients aged 50 years and over than those <50. Several prior research documented a strong association between anemia in diabetic patients with older age [1, 71, 72]. The possible reason for the high prevalence of anemia with increasing age might be chronic diseases/ comorbidities, nutritional disorders such as iron, folate, and vitamin B_12_ deficiency, and systemic inflammation [73–75]. A distinctive hyperinflammatory state is typically seen in at least one-third of elderly anemic patients, especially with chronic kidney disease, cancer, autoimmune disease, and chronic infection [73].

Understandably, most of the studies included in this review reported the prevalence of anemia among type-2 diabetic patients due to its growing burden in South Asia. Patients with type-2 DM were two-fold more likely to develop anemia than patients without DM [76]. The prevalence of anemia was 48% (95% CI: 40.0–56.0) in type-2 DM and 2% in type-1 DM (2%, 95% CI: 0.00–4.00), higher than the evidence in Africa [14]. Other countries such as Brazil, Australia, and the United Kingdom also reported lower anemia prevalence among type-2 diabetic patients than our estimate [77–79]. Several factors might influence the high prevalence of anemia in South Asian diabetic people, such as—poor glycemic control, delayed diagnosis, and poor adherence to treatment and self-care behavior [78, 80, 81]. Only one-third of diabetic patients in South Asia have optimal guideline-recommended glycemic control [82], which may heighten the risk of diabetes-related complications among the vast majority of the diabetic population. Chronic hyperglycemia promotes a hypoxic environment in renal interstitium through abnormal red blood cells, sympathetic denervation of the kidney due to auto-immune neuropathy, oxidative stress, reduced nitric oxide bioavailability, and increased apoptosis of renal tubular cells, which eventually impairs the production of erythropoietin [8, 11]. Hence, many other factors may associate to create such a hypoxic environment that include diabetic nephropathy, chronic inflammation, elevated advanced glycation end products, nutritional deficiency, diabetic neuropathy, and low levels of testosterone [11]. In addition to erythropoietin deficiency, hypo-responsiveness to erythropoietin may occur due to increased glycation of erythropoietin receptors can potentiate the development of anemia [8] in type-2 diabetic patients.

Only one study reported anemia prevalence in patients with gestational DM (GDM), which was 6% (95% CI: 3.00–12.0). Although there is limited evidence, it implies that GDM may be associated with iron disorder, providing a basis for further investigation. The relationship of anemia and GDM is yet to conclusive, however suggested an inverse association. A retrospective case-control study found that pregnant women with iron deficiency anemia had lower risk of developing GDM [83]. Some studies observed a positive correlation between high serum iron and ferritin levels and the risk of developing GDM, possibly linked with increased iron intake during pregnancy [84, 85]. In addition, excess heme iron increases oxidative stress and generates reactive free radicals, thereby contributing to the higher risk of gestational DM [8].

The risk of microvascular complications can be accelerated among diabetic patients in the presence of anemia [13, 86]. We found a higher prevalence of anemia in patients with diabetic complications in South Asia compared to prior evidence [87–90]. Although limited, the pooled estimates from available studies demonstrated a high rate of anemia in patients with diabetic complications, such as neuropathy (68%, 95% CI: 54.0–79.0), nephropathy (49%, 95% CI: 16.0–83.0), and retinopathy (43%, 95% CI: 19.0–67.0). These findings might be explained by the high burden of diabetes and associated risk factors among South Asians [91, 92]. Since diabetes and anemia are both multifactorial, a higher burden of genetic [93, 94], biological, sociodemographic, and lifestyle-related correlates [92], including nutritional disorder [11] among South Asians may predispose the increased risk of co-existing anemia with diabetic complications. The chronicity of DM may also be associated with anemia, as we found higher prevalence of amenia among those who had diabetes 5 years or more. Moreover, studies identified in this review were primarily hospital-based, samples most likely consisting of sicker patients with poor glycemic control or having other associated comorbidities that may result in a higher prevalence of anemia.

In patients with DM, anemia is identified as an independent risk factor for the progression of diabetic microvascular complications [89, 90, 95]. A recent meta-analysis in Ethiopia reported that diabetic patients with anemia were 8.59 times more likely to develop chronic nephropathy than their non-anemic counterparts [1]. Generally, anemia is found to be highly prevalent and more severe in patients with DM at any level of GFR compared to non-diabetic patients [96]. Furthermore, an inverse association was observed between declining GFR and a correspondingly increasing prevalence of anemia in diabetic patients [97]. Hence, functional erythropoietin deficiency and/ or erythropoietin resistance in DM are the plausible reason for developing anemia [11]. Additionally, a hyper-inflammatory state frequently seen in diabetic patients stimulates increased production of pro-inflammatory cytokines such as interleukin-1, interleukin-6, tumor necrosis factor (TNF-α), transforming growth factor, and interferon [8, 14]. These cytokines play a critical role in insulin resistance, vascular complications, decreasing erythropoietin production and efficiency, and promoting apoptosis of immature RBCs, causing a further deficiency of circulating RBCs [98, 99]. In patients with diabetic retinopathy, the risk of anemia mounts up with its progression [89], also associated with tissue hypoxia and ischemia resulting from impaired autoregulation of microvasculature and capillary obstruction [100]. Furthermore, blunted erythropoietin response led by splanchnic autonomic denervation is associated with developing anemia among patients with diabetic neuropathy [11].

Sub-group analysis revealed the highest prevalence of anemia in Pakistan (58%, 95% CI: 40.0–75.0), followed by Bangladesh (53%, 95% CI: 44.0–63.0) and India (43%, 95% CI: 34.0–52.0). However, these interpretations should be used cautiously due to the high heterogeneity marked by *I*^*2*^ statistics. Studies conducted in hospital settings showed a higher pooled prevalence of anemia (46%, 95% CI: 37.0–55.0) compared to studies based on community (16%, 95% CI: 14.0–18.0). Of the hospital-based studies, a small variation in the pooled prevalence of anemia was observed considering the location of recruitment such as In-patient department (IPD) (47%, 95% CI: 5.00–88.0), Both OPD & IPD/unspecified (47%, 95% CI: 34.0–60.0), and out-patient department (OPD) (45%, 95% CI: 37.0–52.0). The huge variation in the pooled estimation of anemia in study settings may occur since the tendency of diabetic patients visiting the hospital are more likely to be in poor health status than patients recruited from the community. Moreover, substantial changes in socioeconomic, lifestyle, and dietary behavior due to rapid urbanization, industrialization, and technological advancements resulting a high burden of metabolic risk factors among south Asians [101], which may contribute to the high prevalence of concurrent anemia and diabetes.

Considering study quality, studies with a low risk of bias reported the prevalence of anemia at 50% (95% CI: 26.0–74.0) and descended in moderate (49%, 95% CI: 31.0–68.0) and high risk of bias (42%, 95% CI: 34.0–51.0). Studies with case-control and cross-sectional design showed somewhat similar prevalence of anemia among diabetic patients while cohort study reported a lower prevalence. However, most studies had serious methodological limitations regarding study design, sampling, and recruitment strategy. Studies with case-control and cohort design did not elaborate on details of recruiting case and control groups in method sections, leaving their studies seriously inconsistent. Furthermore, heterogeneity and variability of pooled estimates as indicated in sub-group analysis may result due to methodological and other population-level differences across the studies. High publication bias in this review emphasizes the lack of overall research evidence that reflects a research gap in the related field. Additionally, inadequate information on the sociodemographic, household, and other modifiable/non-modifiable risk factors limits our ability to assess further potential sources of variances in population groups. Diverse study settings, including a greater number of hospital studies and a few community-based studies, may affect the true burden of anemia within the study population. Since a major proportion of the recruited studies had low quality, it compromised the generalizability of synthesized findings. Despite such limitations, this quantitative synthesis of epidemiological evidence may provide a broader and contextual understanding that can be useful for evaluating the current burden of anemia in diabetic patients and its contributing factors.

The summarized evidence can facilitate establishing country-specific or regional priorities in developing and implementing pragmatic, cost-effective, scalable, evidence-based policies in reducing diabetes mellitus and related complications. Hence, need to focus on the following areas: increasing community awareness, screening of high-risk groups, prioritizing vulnerable groups of people (women, older people, underprivileged, and pregnant women), strengthening primary or community health centers for regular hematological screening, early diagnosis, and timely management, and effective referral system by connecting health centers across different tiers. Finally, intervention studies are required by addressing all modifiable determinants to establish context-specific, cost-effective strategic plans for preventing and managing anemia in diabetes among the South Asian population.

## 5. Conclusion

This review implies that anemia is a critical public health burden among patients with DM in South Asia. These suggest the inclusion of anemia screening into the routine assessment for early diagnosis and management that may deter the progression of diabetes-related complications. However, limited research, high heterogeneity, and low-quality studies necessitate more clinical and epidemiological research following a high scientific protocol to investigate the pathophysiology of anemia among DM patients in South Asia.

## Supporting information

S1 Checklist PRISMA 2020 checklist. (PDF)

S1 Fig Sensitivity analysis (leave one out) of included studies reporting prevalence of anemia among diabetic patients in South Asia. (TIF)

S2 Fig Egger’s test for assessing publication bias. (TIF)

S1 Table Quality assessment of included studies (N = 40). (PDF)

S2 Table Meta-regression of study-characteristics associated with heterogeneity. (PDF)

S1 File Search strategies. (PDF)
