# Correction: Prevalence of anemia in diabetes mellitus in South Asia: A systematic review and meta-analysis

**DOI:** 10.1371/journal.pone.0295777

**Published:** 2023-12-07

**Authors:** Hoimonty Mazumder, Kazi Faria Islam, Farzana Rahman, Easter Protiva Gain, Nobonita Saha, Irfath Sharmin Eva, Md Monir Hossain Shimul, Jyoti Das, M. Mahbub Hossain

There is an error in the article title. The correct title is: Prevalence of anemia among people with diabetes mellitus in South Asia: A systematic review and meta-analysis.

## References

[1] Mazumder H, Islam KF, Rahman F, Gain EP, Saha N, Eva IS, et al. (2023) Prevalence of anemia in diabetes mellitus in South Asia: A systematic review and meta-analysis. PloS ONE 18(5): e0285336. 10.1371/journal.pone.0285336 37163539 PMC10171606

