# Supplementary figures and images for: Prevalence of anemia in diabetes mellitus in South Asia: A systematic review and meta-analysis
Source: PLoS One. 2023 May 10;18(5):e0285336. doi: 10.1371/journal.pone.0285336 (PMC10171606; doi:10.1371/journal.pone.0285336)

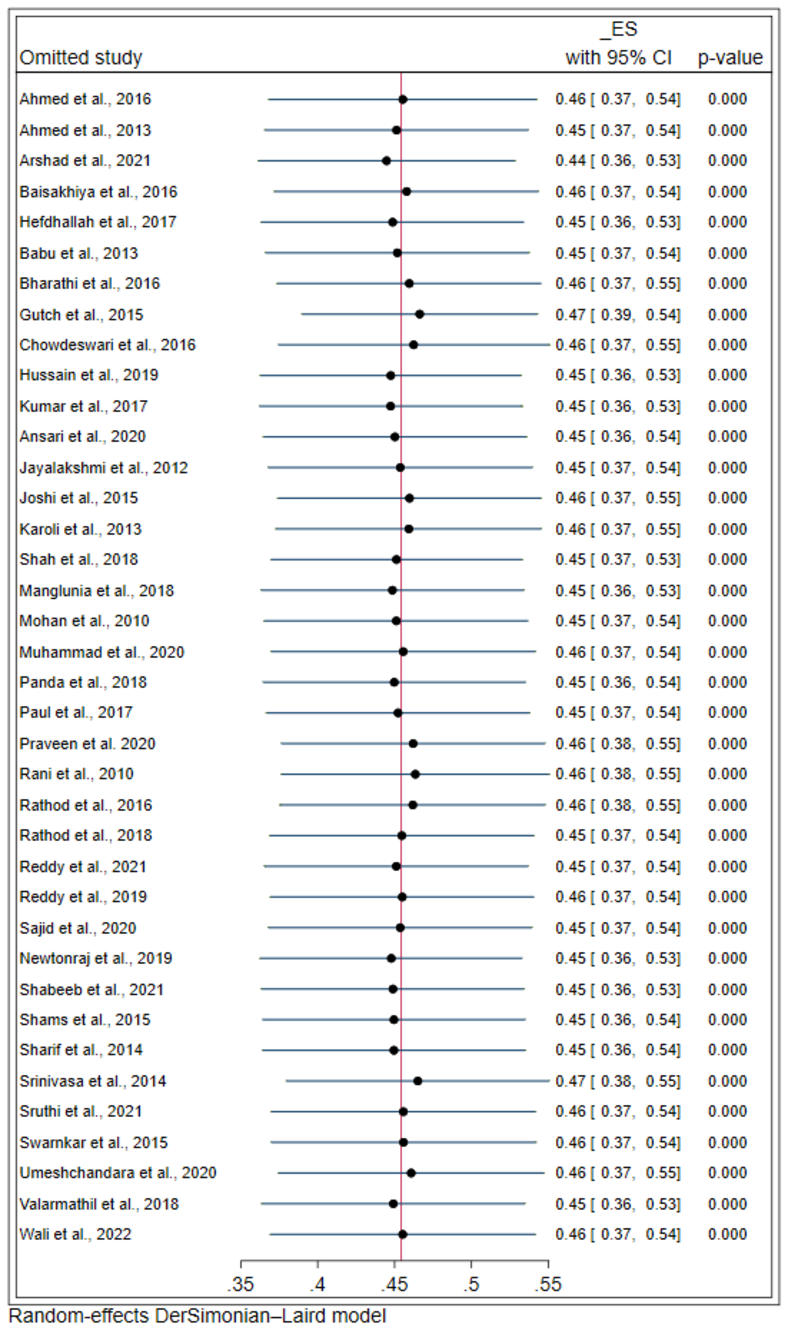

Supplement: S1 Fig — (TIF) [file pone.0285336.s002.tif]

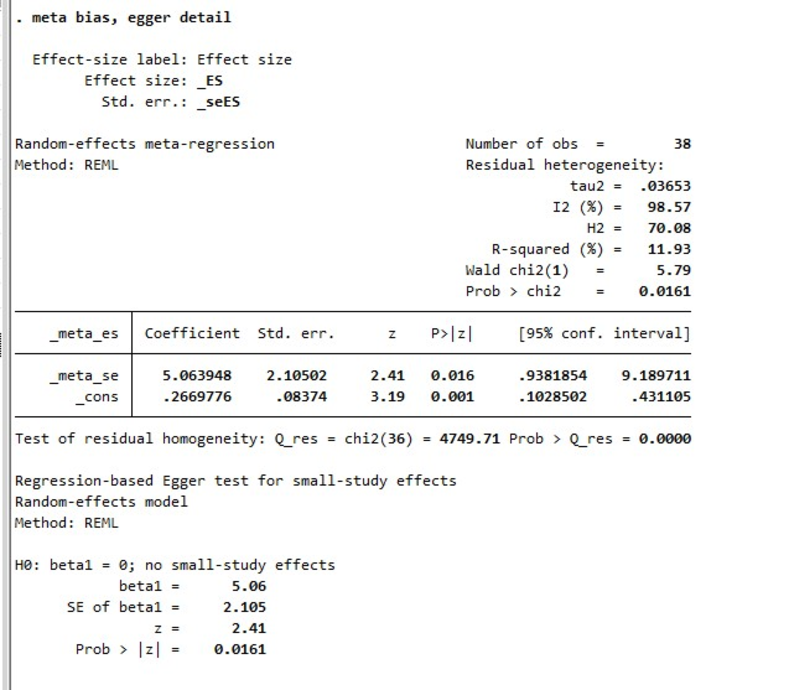

Supplement: S2 Fig — (TIF) [file pone.0285336.s003.tif]
