# Supplementary material for: Prevalence of anemia in diabetes mellitus in South Asia: A systematic review and meta-analysis
Source: PLoS One. 2023 May 10;18(5):e0285336. doi: 10.1371/journal.pone.0285336 (PMC10171606; doi:10.1371/journal.pone.0285336)
Supplement: S1 Table — (PDF) [file pone.0285336.s004.pdf]

S1 Table: Quality assessment of included studies (N = 40).

Author's name and publication year	Q1	Q2	Q3	Q4	Q5	Q6	Q7	Q8	Q9	Total	Final category (in terms risk of bias)
Ahmed et al., 2016	1	1	1	1	0	1	1	1	1	8	Low
Ahmed et al., 2013	0	0	0	1	0	0	U	1	1	3	High
Hefdhallah et al., 2017	1	0	0	0	0	1	1	0	1	3	High
Ansari et al., 2020	1	1	0	0	U	1	1	0	1	5	Medium
Arshad et al., 2021	1	1	1	0	0	1	U	0	1	5	Medium
Babu et al., 2013	0	0	0	1	U	1	1	1	1	5	Medium
Baisakhiya et al., 2016	0	0	0	1	1	1	1	0	1	5	Medium
Bharathi et al., 2016	0	0	0	0	0	1	1	0	1	3	High
Chowdeswari et al., 2016	1	0	1	0	0	1	1	0	1	5	Medium
Gutch et al., 2015	1	0	1	0	0	1	1	0	1	5	Medium
Kumar et al., 2017	0	0	U	1	1	1	1	0	1	5	Medium
Hussain et al., 2019	0	0	1	1	1	1	1	1	1	7	Low

Jayalakshmi et al., 2012	0	1	0	0	0	1	1	0	1	4	High
Joshi et al., 2015	0	0	0	0	U	1	1	0	1	3	High
Karoli et al., 2014	0	0	1	1	1	U	U	0	1	4	High
Shah et al., 2018	1	NA	1	1	1	1	1	0	1	7	Low
Manglunia et al., 2018	0	0	0	0	U	U	U	0	1	1	High
Mohan et al., 2010	0	0	1	1	0	1	1	0	1	5	Medium
Muhammad et al., 2020	0	0	0	0	0	1	1	0	1	3	High
Panda et al., 2018	0	0	0	0	U	U	U	0	1	1	High
Paul et al., 2017	0	0	0	0	0	U	U	0	1	1	High
Praveen et al., 2020	0	U	0	0	1	1	1	1	1	5	High
Rahman et al., 2010	1	1	1	1	1	1	1	1	1	9	Low
Rahman et al., 2012	1	1	1	1	1	1	1	1	1	9	Low
Rani et al., 2010	1	1	1	1	1	1	1	1	1	9	Low
Rathod et al., 2016	0	U	0	0	U	1	1	0	1	3	High

Rathod et al., 2018	0	U	0	0	0	U	U	0	1	1	High
Reddy et al., 2021	0	1	0	1	0	1	U	1	1	5	Medium
Reddy et al., 2019	0	0	0	0	U	1	0	0	1	1	High
Sajid et al., 2020	0	1	0	0	1	1	1	1	1	6	Medium
Newtonraj et al., 2019	1	1	1	1	1	1	1	1	1	9	Low
Shabeeb et al., 2021	0	0	0	0	U	0	0	0	1	1	High
Shams et al., 2015	0	1	0	1	U	1	1	1	1	6	Medium
Sharif et al., 2014	0	0	0	0	U	1	U	0	1	2	High
Srinivas et al., 2014	0	0	0	0	NA	0	U	0	1	1	High
Sruthi et al., 2021	0	0	0	0	U	1	1	0	1	3	High
Swarnkar et al., 2015	0	0	0	0	0	1	1	0	1	3	High
Umeshchandra et al., 2021	0	0	0	0	0	0	0	0	1	1	High
Valarmathil et al., 2018	0	0	0	0	0	0	0	0	1	1	High
Wali et al., 2022	0	0	0	0	U	1	1	0	1	3	High

U = Unclear, NA = Not applicable
