# Supplementary material for: Prevalence of anemia in diabetes mellitus in South Asia: A systematic review and meta-analysis
Source: PLoS One. 2023 May 10;18(5):e0285336. doi: 10.1371/journal.pone.0285336 (PMC10171606; doi:10.1371/journal.pone.0285336)
Supplement: S2 Table — (PDF) [file pone.0285336.s005.pdf]

S2 Table: Meta-regression of study-characteristics associated with heterogeneity.

Variables	Coefficient	p-value
Country	0.102	0.156
Mean age	0.008	0.002
Sample size	-0.05	0.439
Type of DM	0.077	0.281
Study design	-0.003	0.970
Study settings	-0.053	0.720
Risk of bias	-0.044	0.332
