# Supplementary material for: Prevalence of anemia in diabetes mellitus in South Asia: A systematic review and meta-analysis
Source: PLoS One. 2023 May 10;18(5):e0285336. doi: 10.1371/journal.pone.0285336 (PMC10171606; doi:10.1371/journal.pone.0285336)
Supplement: S1 File — (PDF) [file pone.0285336.s006.pdf]

Search strategies in selected databases

Detail search strategy in Medline:

Name of database	Keywords were searched in Medical Subject Headings (MeSH) and title and abstract
Medline	1. "Anemia"
	2. “Anaemia”
	3. “hematological parameters”
	4. “red blood cell parameters”
	5. 1 OR 2 OR 3 OR 4
	6. “Diabetes Mellitus”
	7. “Diabetic”
	8. “Diabetic patient”
	9. “Glucose tolerance test”
	10. “Glucose intolerance”
	11. “Hyperglycemia”
	12. “Blood glucose”
	13. “Gestational Diabetes”
	14. “Impaired glucose tolerance”
	15. “Diabetes*”
	16. 6 OR 7 OR 8 OR 9 OR 10 OR 11 OR 12 OR 13 OR 14 OR 15
	17. “Prevalence”
	18. “Prevalence*”
	19. “Incidence”
	20. “associated factors”
	21. “Determinant factors”
	22. “Determina*”
	23. “Epidemiology”
	24. “Epidemiology*”
	25. “Disease burden”
	26. “Frequency*”
	27. “Magnitude”
	28. 17 OR 18 OR 19 OR 20 OR 21 OR 22 OR 23 OR 24 OR 25 OR 26 OR 27
	29. “Afghan*”
	30. “Bangladesh*”
	31. “Bhutan*”
	32. “India*”
	33. “Maldiv*”
	34. “Nepal*”
	35. “Pakistan*”
	36. “Sri Lanka*”
	37. “South Asia”
	38. 29 OR 30 OR 31 OR 32 OR 33 OR 34 OR 35 OR 36 OR 37
	39. 5 AND 16 AND 28 AND 38

<u>Detail search strategy in remaining four databases:</u>

Name of database	Keywords were searched for retrieving literature
APA PsycInfo, Academic Search Ultimate, CINAHL, and Web of Sciences	1. "Anemia"
	2. “Anaemia”
	3. “hematological parameters”
	4. “red blood cell parameters”
	5. 1 OR 2 OR 3 OR 4
	6. “Diabetes Mellitus”
	7. “Diabetic”
	8. “Diabetic patient”
	9. “Glucose tolerance test”
	10. “Glucose intolerance”
	11. “Hyperglycemia”
	12. “Blood glucose”
	13. “Gestational Diabetes”
	14. “Impaired glucose tolerance”
	15. “Diabetes*”
	16. 6 OR 7 OR 8 OR 9 OR 10 OR 11 OR 12 OR 13 OR 14 OR 15
	17. “Prevalence”
	18. “Prevalence*”
	19. “Incidence”
	20. “associated factors”
	21. “Determinant factors”
	22. “Determina*”
	23. “Epidemiology”
	24. “Epidemiology*”
	25. “Disease burden”
	26. “Frequency*”
	27. “Magnitude”
	28. 17 OR 18 OR 19 OR 20 OR 21 OR 22 OR 23 OR 24 OR 25 OR 26 OR 27
	29. “Afghan*”
	30. “Bangladesh*”
	31. “Bhutan*”
	32. “India*”
	33. “Maldiv*”
	34. “Nepal*”
	35. “Pakistan*”
	36. “Sri Lanka*”
	37. “South Asia”
	38. 29 OR 30 OR 31 OR 32 OR 33 OR 34 OR 35 OR 36 OR 37
	39. 5 AND 16 AND 28 AND 38
